# Hippocampal volume mediates the relationship of parental rejection in childhood with social cognition in healthy adults

**DOI:** 10.1038/s41598-023-46512-2

**Published:** 2023-11-06

**Authors:** Marino Kawamoto, Haruto Takagishi, Toru Ishihara, Shunsuke Takagi, Ryota Kanai, Genichi Sugihara, Hidehiko Takahashi, Tetsuya Matsuda

**Affiliations:** 1 Department of Psychiatry and Behavioral Sciences, Tokyo Medical and Dental University Graduate School, Tokyo, Japan; 2 Tamagawa University Brain Science Institute, Tokyo, Japan; 3 Graduate School of Human Development and Environment, Kobe University, Kobe, Japan; 4 Araya Inc., Tokyo, Japan; 5 Center for Brain Integration Research, Tokyo Medical and Dental University, Tokyo, Japan

**Keywords:** Social neuroscience, Cognitive neuroscience

## Abstract

Childhood abuse reduces hippocampal and amygdala volumes and impairs social cognition, including the ability to recognize facial expressions. However, these associations have been studied primarily in individuals with a history of severe abuse and psychiatric symptoms; researchers have not determined whether these associations can also be observed in healthy adults. In the present study, we analyzed data from 400 healthy adults (208 men and 192 women) at Tamagawa University. Parental rejection reflecting childhood abuse was assessed using the short form of Egna Minnen Beträffande Uppfostran, while social cognition was assessed using the “Fake Smile Detection Task.” Hippocampal and amygdala volumes were extracted from T1-weighted magnetic resonance imaging data using FreeSurfer. We found that greater parental rejection resulted in smaller hippocampal and amygdala volumes and poorer performance in the Fake Smile Detection Task. Structural equation modeling analysis supported the model that hippocampal volume mediates maternal rejection effect on performance on the Fake Smile Detection Task, with involvement of the amygdala. These findings are in line with the structural and functional connectivity found between the hippocampus and amygdala and their joint involvement in social cognition. Therefore, parental rejection may affect hippocampal and amygdala volumes and social cognitive function even in symptom-free adults.

## Introduction

Childhood abuse affects physical and mental development and increases the risk of various mental problems in adulthood, including depression, anxiety disorders, alcohol use disorders, antisocial behavior, and personality disorders^1–4^. Abuse is mainly classified as physical abuse, emotional abuse, sexual abuse, and neglect. A large US study reported that 24.9% individuals aged 0–17 years experienced abuse in their lifetime, with 9.8%, 14.5%, 2%, and 11.8% experiencing physical abuse, emotional abuse, sexual abuse, and neglect, respectively, with overlap in each abuse subcategory^5^. Another study using a large database of US health maintenance organizations reported that all these types of abuse have adverse effects on mental health. Furthermore, a study showed that emotionally abusive family environments had strong negative effects on mental symptoms^6^. Rejective parenting attitudes, where parents ignore their children’s needs, belittle them in front of others, or punish them excessively without justification, have been associated with adolescent depression^7^.

Abuse affects brain development, causing sustained structural brain changes by interfering with normal neurodevelopment. For example, in abused or maltreated children, there is a decrease in the thickness and surface area of the cerebral cortex, as well as a decrease in hippocampal and amygdala volumes, during childhood and early adolescence^8–13^. These structural changes in the brain may increase the risk of mental health problems, with the hippocampus and amygdala playing important roles in their etiology. The hippocampus plays an important role in not only memory and learning but also empathy for others and future imagination; moreover, it is involved in regulating negative feedback in the hypothalamic–pituitary–adrenal axis, which controls cortisol production in response to stressors, thus regulating vulnerability to mental disorders^14–23^. Moreover, because the amygdala is responsible for threat judgment and emotional processing, amygdala reactivity to negative stimuli is associated with anxiety and depression^24,25^. It has been reported that a reduction in the hippocampal and amygdala volumes could contribute to mental health problems after exposure to abuse^26^.

Abused children have impaired social cognitive function^27,28^. Social cognitive function refers to an individual’s ability to use information in social contexts to explain and predict behavior, and it is associated with strong social skills^29^. It is considered to develop in the context of nurturing interactions experienced during early childhood^30^. The maturity of social cognitive function can be assessed using several tasks, including facial expression recognition tasks, which measure the ability to recognize others’ facial expressions and accurately read emotions from facial expressions, and theory of mind tasks, which assess the ability to understand others’ thoughts^30,31^. Among facial expressions, genuine smiles are a strong social cue of a person’s willingness to cooperate, and the ability to distinguish between genuine and fake smiles is thought to help individuals make appropriate choices regarding their relationships with others^32,33^. Most children with typical development can recognize major facial expressions and complete the false-belief task by school age, which is impeded in abused children^34–37^.

Only a few studies have investigated social cognitive function in adults who have experienced childhood abuse. Nonetheless, the aforementioned findings and a report of decreased performance in expression recognition tasks among healthy adults after laboratory-induced attachment-related stress suggest that childhood abuse impairs social cognitive function in adulthood^38^.

The relationship between brain structural alterations induced by childhood abuse and social cognitive function, especially in individuals without overt mental health problems, remains unclear. Therefore, the aim of this study was to examine whether hippocampal and amygdala volumes mediate the effects of abuse on fake smile detection, which is important for judging the trustworthiness of others in social life.

## Results

### Descriptive statistics

The participants comprised 400 adults (192 women) aged 20–59 years (mean [SD] 40.8, [10.4]). The mean bilateral total hippocampal volume was 8263 mm^3^ (SD: 825 mm^3^), while the mean bilateral total amygdala volume was 3542 mm^3^ (SD: 491 mm^3^). The mean intracranial volume was 1,425,354 mm^3^ (SD: 146,582 mm^3^). Table 1 summarizes the demographic and clinical variables of the participants.

**Table 1:** Distribution of study variables.

Variables	N	Mean	SD	Frequency (%)	K-S test of normality (Lilliefors test) (*p*)
Age (years)	400	40.6	10.4		< .001
Sex					< .001
Male	208			52.0	
Female	192			48.0	
IQ	400	99.6	12.8		.002
Brain MRI data					
Bilateral total hippocampal volume (mm^3^)	400	8263	825		.200
HpVR (%)	400	.583	.062		.189
Bilateral total amygdala volume (mm^3^)	400	3542	491		.141
AmVR (%)	400	.250	.031		.200
Intracranial volume (mm^3^)	400	1,425,354	146,582		.200
s-EMBU					
Parental rejection	400	1.34	.40		< .001
Paternal rejection	400	1.34	.46		< .001
Maternal rejection	400	1.35	.45		< .001
Fake Smile Detection Task					
Number of correct answers	400	13.8	2.5		< .001

Intellectual evaluation was conducted using the IQ test “Kyoto University NX-15”^39^.

*HpVR* hippocampal volume:intracranial volume ratio, *AmVR* amygdala volume:intracranial volume ratio, *s-EMBU* short form of Egna Minnen Beträffande Uppfostran, *MRI* magnetic resonance imaging, *SD* standard deviation.

### Correlation analyses

**Figure 1:**
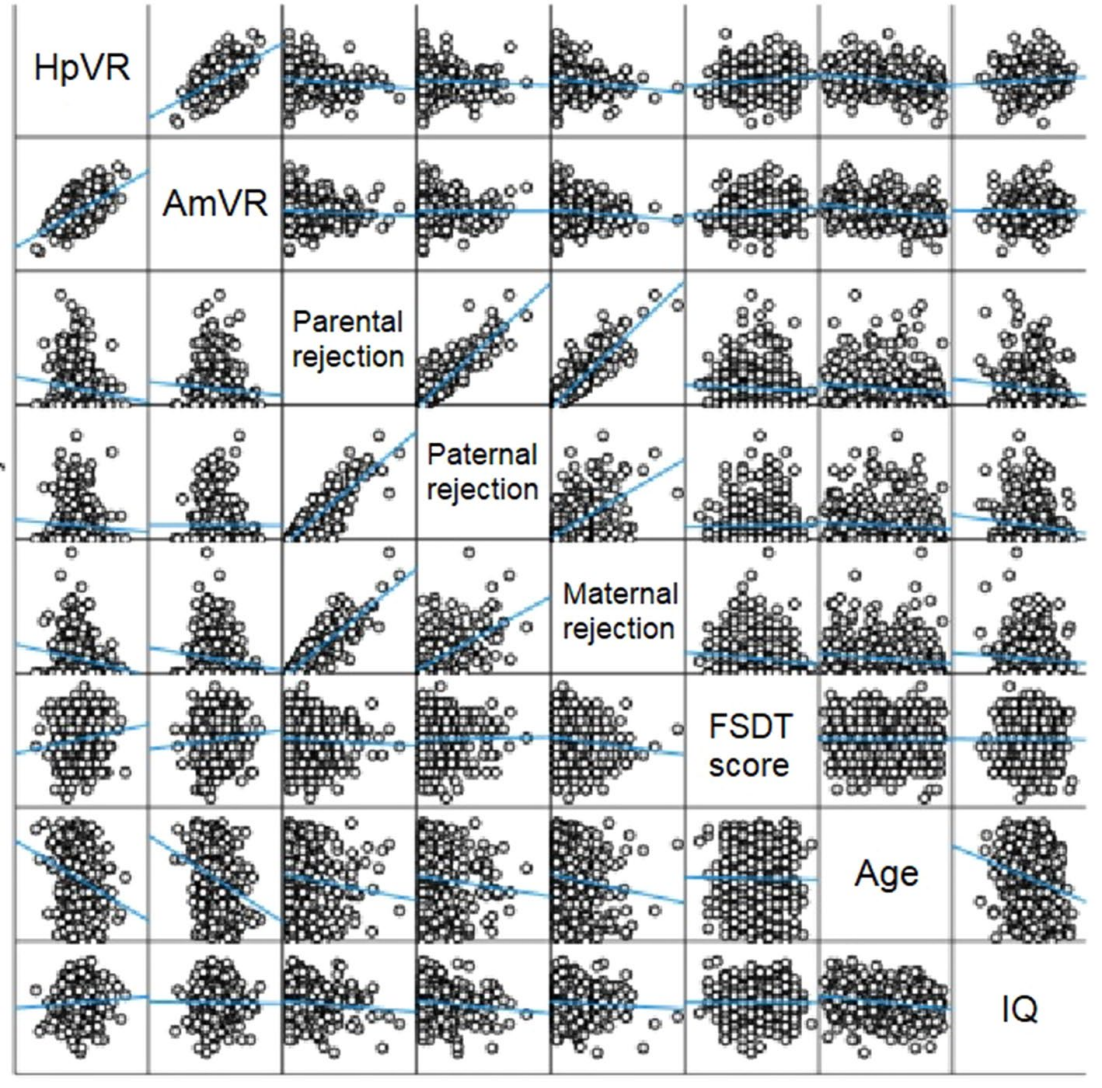
Scatterplot matrix. The straight lines indicate linear approximations. HpVR, hippocampal volume:intracranial volume ratio; AmVR, amygdala volume:intracranial volume ratio; FSDT, Fake Smile Detection Task.

A scatterplot matrix for HpVR, AmVR and EMBU rejection scores, Fake Smile Detection Task score, and the covariates age, sex and IQ are displayed in (Fig. 1). Linear approximation lines are shown overlaid with the scatterplot matrix. Positive correlations were demonstrated between hippocampal and amygdala volumes and between parental rejection and paternal and maternal rejection.

EMBU rejection scores and Fake Smile Detection Task scores are not normally distributed; therefore, correlations between each variable were examined using Spearman's rank correlation coefficient (Table 2). The participants' age showed significant negative correlations with hippocampal and amygdala volumes and parental/paternal/maternal rejection scores (Rs =  − 0.224, *p* = . < 001; Rs =  − 0.242, *p* = . < 001; Rs =  − 0.138, *p* = 0.006; Rs =  − 0.100, *p* = 0.046; Rs =  − 0.141, *p* = 0.005, respectively) but no significant correlations with the performance of the Fake Smile Detection Task (Rs =  − 0.015, *p* = 0.770). The IQ was negatively correlated with age only (Rs =  − 0.194, *p* = . < 001), but not significantly with other variables. The effect of sex was examined using the Mann–Whitney U test, which suggested a possible sex difference in hippocampal and amygdala volumes (Z =  − 2.093, *p* = 0.036; Z = 2.604, *p* = 0.009, respectively), but no significant difference in rejection scores and Fake Smile Detection Task scores.

**Table 2:**
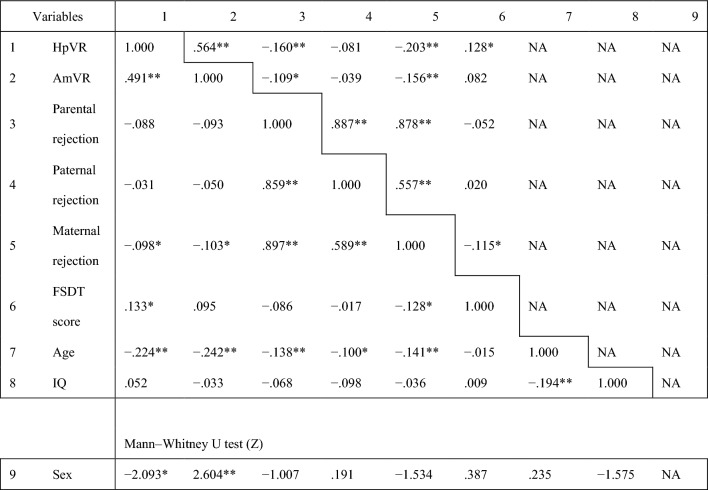
Correlation matrix.

The lower left side of the table displays Spearman's correlation coefficients. The upper right side of the table displays Spearman's partial correlation coefficients adjusted for age, sex, and IQ.

*HpVR* hippocampal volume:intracranial volume ratio, *AmVR* amygdala volume:intracranial volume ratio, *FSDT* fake smile detection task, *NA* not applicable.

**p < .01; *p < .05.

Spearman's rank correlation coefficients, adjusted for age, sex, and IQ as covariates, are shown in the upper right corner of Table 2.

We first examined the correlation between the rejection scores and hippocampal/amygdala volumes. Parental rejection was significantly negatively correlated with hippocampal and amygdala volumes (Rs =  − 0.160, *p* = 0.001; Rs =  − 0.109, *p* = 0.030, respectively), and maternal rejection was also significantly negatively correlated with hippocampal and amygdala volumes (Rs =  − 0.203, *p* = . < 001, Rs =  − 0.156, *p* = 0.002, respectively), while paternal rejection showed no significant correlation with hippocampal and amygdala volumes (Rs =  − 0.081, *p* = 0.108, Rs =  − 0.039, *p* = 0.442, respectively).

Regarding the correlation between rejection scores and Fake Smile Detection Task scores, only maternal rejection had a significant negative correlation with the Fake Smile Detection Task scores (Rs =  − 0.115, *p* = 0.022), while neither the parental rejection nor the paternal rejection showed a significant correlation (Rs =  − 0.052, *p* = 0.298; Rs = 0.020, *p* = 0.690, respectively).

Lastly, we examined the correlation between hippocampal and amygdala volumes and performance on the Fake Smile Detection Task. The Fake Smile Detection Task score had a significant positive correlation with hippocampal volume (Rs = 0.128, *p* = 0.010), but not with amygdala volume (Rs = 0.082, *p* = 0.103).

We examined the effects of paternal and maternal rejection, respectively, and found that hippocampal and amygdala volumes and performance on the Fake Smile Detection Task scores were significantly correlated only with maternal rejection. However, the Wilcoxon signed rank sum test results indicated no significant difference between paternal and maternal rejection scores (Z = 0.503, *p* = 0.615).

### Structural equation modeling analysis

**Figure 2:**
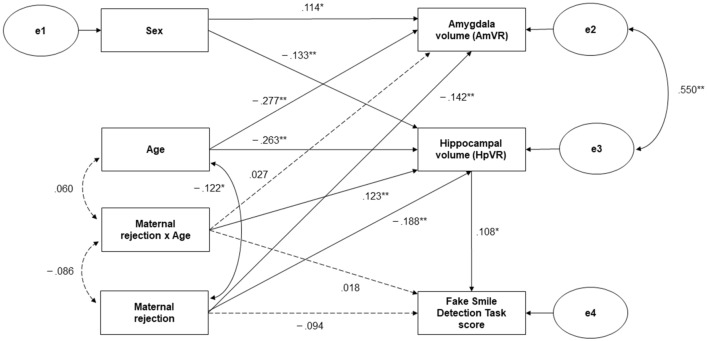
Structural Equation Model: Hippocampal volume as the mediator of maternal rejection and Fake Smile Detection Task score. All path coefficients except the correlation coefficient (r) are standardized. The 95% bootstrapped confidence intervals are based on 10,000 iterations. Solid lines indicate significant relations. Dotted lines indicate nonsignificant relations. e1–4 indicate errors. **p < .01; *p < .05.

Based on the results of the correlation analysis, a structural equation modeling (SEM) analysis was performed to determine whether hippocampal volume mediates the effects of maternal rejection on social cognitive function. We controlled for age and sex with reference to the aforementioned results. An interaction term between maternal rejection and age was added to the model, as we hypothesized that the negative effects of maternal rejection on brain structure and function would diminish with age. We also assumed structural and functional associations between the hippocampus and amygdala, and added a covariance path for both. The model results are shown in Fig. 2, along with the standardized coefficients for each path.

The model provided statistically acceptable accounts of the data, chi-squared (6) = 2.374, *p* = 0.882, comparative fit index (CFI) = 1.000, root mean square error of approximation (RMSEA) = 0.000, and Akaike information criterion (AIC) = 46.374. Estimates for each path are reported in Supplementary Table S1 online.

Subsequently, the mediating effects of hippocampal volume on the relationship between maternal rejection and performance in the Fake Smile Detection Task were analyzed. We found that the total effect of maternal rejection on the Fake Smile Detection Task score was significant (β =  − 0.114, *p* = 0.014). Moreover, the indirect effect (hippocampal volume-mediated) of maternal rejection on the Fake Smile Detection Task score via hippocampal volume was significant (β =  − 0.020, *p* = 0.046), while the direct effect of maternal rejection on the Fake Smile Detection Task score was non-significant (β =  − 0.094, *p* = 0.062). Thus, a mediating effect of the hippocampal volume was found. The ratio of indirect effect to total effect was 17.5% (Table 3). The correlation coefficient between the hippocampus and amygdala was significant (r = 0.550, *p* = . < 001), suggesting that the hippocampus and amygdala functions jointly on the mediation of the hippocampus.

**Table 3:** Effects of maternal rejection on the Fake Smile Detection Task score via hippocampal volume in SEM results.

Relationship	B	SE	β	p	Bootstrapped 95% CI
Total effect	− .637	.269	− .114	.014*	[− 1.168, − .131]
Direct effect	− .524	.281	− .094	.062	[− 1.059, .006]
Indirect effect	− .114	.065	− .020	.046*	[− .266, − .001]

The 95% bootstrapped confidence intervals (CIs) are based on 10,000 iterations.

*SEM* structural equation modeling, *B* observed coefficients, *β* standardized coefficients.

*p < .05.

**Figure 3:**
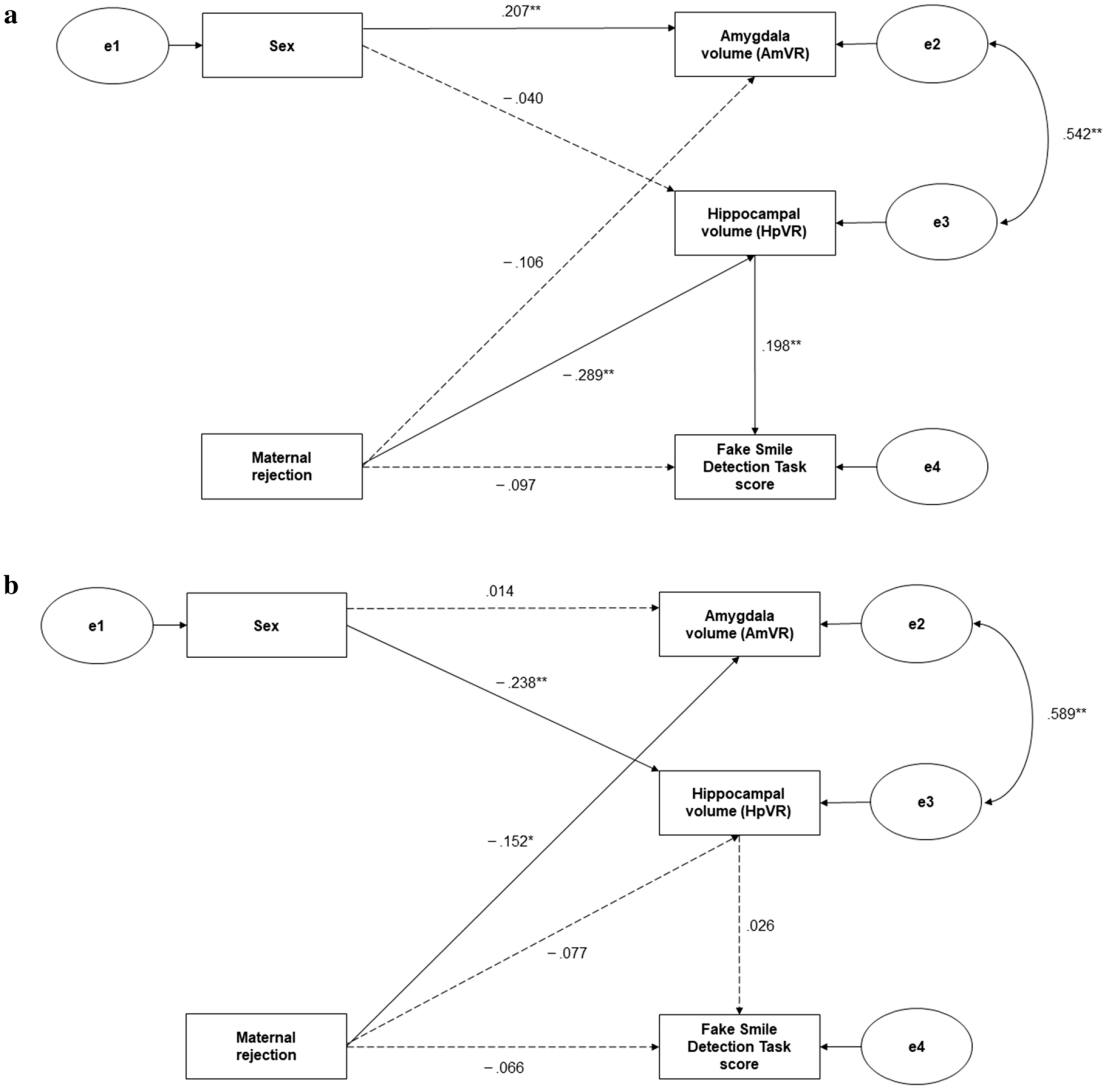
Structural Equation Model: Post hoc analysis for younger (**a**) and older (**b**) groups. All path coefficients except the correlation coefficient (r) are standardized. The 95% bootstrapped confidence intervals are based on 10,000 iterations. Solid lines indicate significant relations. Dotted lines indicate nonsignificant relations. e1–4 indicate errors. **p < .01; *p < .05.

The significant interaction term between maternal rejection and age on hippocampal volume (β = 0.123, *p* = 0.009) suggests that stronger maternal rejection reduces hippocampal volume, but the effect is stronger at younger ages. Furthermore, the mediating effect of hippocampal volume on maternal rejection and performance on the Fake Smile Detection Task may also be stronger at younger ages. To test this hypothesis, a post hoc analysis was conducted with two groups of participants. The mean and median ages of all participants were 40.63 and 41.00 years, respectively. Therefore, using the mean age, we divided the participants into a younger group (< 41 years, N = 197) and an older group (> = 41 years, N = 203) and performed SEM for each group. The model for SEM in the post hoc analysis followed the model of the main analysis, but with the removal of variables related to age, given that the groups were compared by ages. Each model and the standardized path coefficients obtained are shown in Fig. 3.

Both of the models provided statistically acceptable accounts of the data. For the younger group, chi-squared (3) = 3.179 (*p* = 0.365), CFI = 0.998, RMSEA = 0.017, and AIC = 27.179, and for the older group, chi-squared (3) = 0.823 (*p* = 0.844), CFI = 1.000, RMSEA = 0.000, and AIC = 24.823. Estimates for each path are reported in Supplementary Table S2 online.

In the post hoc analysis, the main result was supported in the younger group, but not in the older group. More specifically, in the younger group, the severity of maternal rejection was correlated with smaller hippocampal volume, which in turn was correlated with lower performance on the Fake Smile Detection Task. The indirect effect mediated by hippocampal volume was also significant, predicting 37.0% of the total effect (Table 4).

**Table 4:** Effect of maternal rejection on Fake Smile Detection Task scores via hippocampal volume on younger (a) and older (b) groups (post hoc SEM results).

Relationship	B	SE	β	*p*	Bootstrapped 95% CI
(a)					
Total effect	− .859	.369	− .154	.030*	[− 1.561, − .100]
Direct effect	− .540	.398	− .097	.172	[− 1.320, .239]
Indirect effect	− .319	.143	− .057	.009**	[− .119, − .015]
(b)					
Total effect	− .383	.379	− .068	.273	[− 1.199, .278]
Direct effect	− .371	.378	− .066	.291	[− 1.187, .289]
Indirect effect	− .011	.045	− .002	.500	[− .153, .049]

The 95% bootstrapped confidence intervals (CIs) are based on 10,000 iterations.

*SEM* structural equation modeling, *B* observed coefficients, *β* standardized coefficients.

**p < .01; *p < .05.

## Discussion

We found that parental rejection is associated with smaller hippocampal and amygdala volumes in healthy adults. Additionally, the effect of maternal rejection was stronger than the effect of paternal rejection. The association between maternal rejection and hippocampal volume was more evident in younger ages. Moreover, maternal rejection was associated with poorer performance in the Fake Smile Detection Task, and this relationship was found to be mediated by hippocampal volume and the effect being stronger in younger participants.

Abuse is known to reduce the regional brain volume, which could be strongly influenced by stress^13^. The relationship between brain volume reduction and stress can be explained by an excessive stress response in the endocrine system, which affects neurons rich in glucocorticoid receptors^40^. Rat studies have shown a reduced number of dendrites in hippocampal pyramidal cells after exposure to stress during childhood^41^. These effects are remarkable during the developmental period owing to high stress sensitivity in the central nervous system during neurodevelopment. In humans, the hippocampus is sensitive to stress at the age of 2–3 years and 10–14 years^10^. Therefore, exposure to stress, especially during this period, could reduce hippocampal volume.

Previous studies have shown that childhood abuse reduces hippocampal volume, and our results are consistent with these findings^42^. In our study, these effects were more evident in younger individuals. The negative relationship between age and the effect of rejection has two possible explanations. First, the volume difference between individuals with and without rejection may be smaller at older ages given the aging-related physiological volume reduction of the hippocampus. Second, the adverse effects of abuse may be influenced by later-life environmental changes, including parental separation, employment, and marriage.

Additionally, parental rejection was associated with reduced amygdala volume. The amygdala is involved in emotional cognition and memory processes^43,44^. Because humans use their past personal memories to contextualize their current environment, a strong bias toward negative effects in memory may induce excessive negative effects even for neutral events. In individuals with high rejection scores, a strong negative bias in memory can stress the amygdala, which, in turn, may be associated with its smaller volume.

There have been inconsistent reports regarding the relationship between childhood stress and amygdala volume. For example, rat studies have shown that chronic intense stress increases the number of dendrites of pyramidal cells in the basal part of the amygdala^41^. In humans, there are increased amygdala volumes in children raised by depressed mothers and foster parents^11,45^. In contrast, patients with post-traumatic stress disorder secondary to abuse have been reported to have unchanged or decreased amygdala volumes^8,46^. These inconsistent reports could be attributed to differences in the characteristics of the participants, including age and the presence of mental illness.

Abused and maltreated children present with impaired social cognitive function^47^. For example, abused children have difficulty correctly identifying emotional expressions as well as recognizing the beliefs and purposes that characterize others’ behavior^36,37^. This is because, during infancy, children learn from their parents about facial expressions and the relationship of facial expressions with emotions/behaviors; accordingly, they form a basic model for understanding others^47^. Here, if the emotions directed by parents are unstable or biased toward negative emotions, including anger or rejection, children may not appropriately learn emotional expressions. Accordingly, they may continue using this biased model of understanding others’ emotions even until adulthood. Consistent with these findings, we found that maternal rejection in childhood was associated with poorer performance in the Fake Smile Detection Task. An additional unique feature is that we used a video-clip-based task, which could facilitate better detection of difficulties in social cues in daily life since it is performed in a more real-life setting than tests involving static facial expressions.

Performance in facial expression recognition tasks is related to amygdala function. Bilateral amygdala damage leads to impaired recognition of fearful and sad faces^48–50^. A number of studies have focused on facial expression recognition and amygdala volume, reporting a negative correlation between left amygdala volume and accuracy in recognizing fearful facial expressions, as well as a significant effect of amygdala volume on the perception of sad images^51–53^. Functional magnetic resonance imaging (MRI) studies have reported increased amygdala activity in response to fear and joy while performing facial expression recognition tasks^54–56^. Accordingly, we predicted that performance in the Fake Smile Detection Task would be associated with amygdala volume. However, we found that the relationship between rejection and performance in the Fake Smile Detection Task was mediated by hippocampal volume in correlation with amygdala volume. These results could be attributed to several reasons. First, it is possible that the Fake Smile Detection Task was processed by a network centered on the hippocampus, which may have been associated with smaller hippocampal volume, indicating a functional change in hippocampal neurons. The Fake Smile Detection Task measures the ability to read and judge the social intent of a smile, rather than measuring the ability to distinguish between the emotions of a smile and a straight face, as observed in conventional facial expression recognition tasks. This can be described as a mentalizing process. Mentalizing is associated with memory referencing processes via the hippocampus, and the Fake Smile Detection Task may also be processed by this mechanism^57,58^. Some fMRI studies suggest that the hippocampus plays an important role in eliciting and experiencing social emotions by showing extensive connectivity and strong activation to cortical systems involved in social cognition and self-processing, including the anterior insula, anterior cingulate cortex, superior temporal sulcus, left middle temporal gyrus, prefrontal cortex, and pre-motor cortex^59^. Performances in the Fake Smile Detection Task were not associated with IQ; this suggests that hippocampal volume changes associated with rejection are uniquely related to social cognition.

Second, the hippocampus and amygdala are involved in social cognitive function. There are bidirectional neurofibrillary contacts between the hippocampus and amygdala^60,61^. Additionally, the hippocampus and amygdala are jointly involved in fear conditioning, emotional memory, and social trait/trustworthiness judgments^62,63^. It has been reported that not only the amygdala but also the hippocampus is involved in facial trait detection^64^. Accordingly, the hippocampus and amygdala may be jointly involved in social facial expression recognition. Since the hippocampus is more prone to stress-induced volume changes than the amygdala, the hippocampal effects were stronger in the correlation and SEM analysis.

Third, although the hippocampal volume is a sensitive indicator of abuse-related brain dysfunction, it may not be the site central to abuse-induced social cognitive dysfunction, that is, it may only exert indirect effects, including controlling the function of other responsible sites. This study also found a direct effect from maternal rejection to impaired social cognitive function that was not mediated by hippocampal volume. In addition to our volumetric assessment, functional imaging analysis could allow for better data interpretation.

The mediating effect of hippocampal volume was found stronger in younger adults, which could be attributed to the normalization of memory distortions applied in facial expression recognition with the decrease in the influence of parental rejection as the range of social activities expands during adulthood. Since we included healthy adults without a history of psychiatric treatment, our results suggest their resilience to the adversity of a rejecting environment during childhood. Finally, we found that compared with paternal rejection, maternal rejection was associated with decreased hippocampal and amygdala volumes as well as social cognitive function. Although differences in the effects of paternal and maternal abuse on children are not yet fully known, previous research has shown that maternal physical and emotional abuse during adolescence has a greater impact on the subsequent development of depression than does paternal abuse, and our results are consistent with this theory^65^. This could be attributed to the fact that Japanese mothers have more contact time with their children; accordingly, they are considered to have a greater influence on their children’s care. Although this retrospective study could not determine the contact time between children and parents, most participants’ mothers were full-time housewives. Therefore, it was assumed that they were primary caregivers.

This study had a few limitations. First, we used the s-EMBU, which is a retrospective method for abuse surveys. Prior studies have reported no significant differences in EMBU scores before and after hospitalization for depressed patients and that EMBU scores remained correlated during test-retests at 6-month intervals, suggesting that the EMBU is stable over time and across mental states^66–68^. However, since our participants were between 20 and 50 years of age, the responses of the older participants could have been affected by recall bias. In this study, the correlation coefficient Rs between s-EMBU rejection score and age was weak but statistically significant. This could be one of the reasons why the effect of rejection was weaker in the older age groups. Second, since this was a retrospective study, we could not directly determine the causal relationships among abuse, brain structure, and social cognitive function. Future studies on the same participants are warranted to validate our results.

In conclusion, we found that parental rejection negatively correlated with hippocampal and amygdala volumes as well as performance in the Fake Smile Detection Task. Hippocampal volume mediates the effect of maternal rejection on performance in the Fake Smile Detection Task in relation with amygdala volume, especially in young adults. Our findings suggest that parental rejection affects hippocampal and amygdala volumes as well as social cognitive function in adults without apparent psychiatric symptoms. These results emphasize, once again, the significant impact of abuse on mental health. The causal relationship between hippocampal damage and social cognitive decline can be more strongly discussed by conducting fMRI assessments in a prospective study. If future research can find protective factors for social cognitive decline in rejection survivors, it will directly contribute to the mental health of many people.

## Methods

### Data collection

We used a database constructed in a large-scale research project (Neuro-Psychological and Socio-Institutional Foundations of Pro-Social Behavior, http://www.human-sociality.net/english/) involving healthy residents within a 15-km radius of Machida City, a suburb of Tokyo, Japan. The project aimed to identify the psychological and biological characteristics underlying human prosociality (cooperation, empathy, reciprocity, and fairness) and the process of its expression. Participants in the database were recruited by distribution of brochures to approximately 180,000 households in the target area between March and May 2012. From the 1670 people who voluntarily offered to participate, a total of 600 men and women aged 20–59 years were randomly selected and included in the database [75 men and 75 women in 10-year age groups (20 s, 30 s, 40 s, and 50 s)]. The presence or absence of a history of abuse was not considered during recruitment. The survey was conducted ten times between 2012 and 2018, each at several-month intervals, and data regarding demographic characteristics such as age, sex, height, and weight; economic, cognitive, and psychological test results; salivary testosterone levels; salivary oxytocin levels; genetic results; and head magnetic resonance imaging (MRI) were collected. Although some of those data have already been published (see Supplementary Table S3 online), to our knowledge, this is the first study to examine the relationship between perceived nurturance attitude during childhood and brain structure. We included the following data in our analyses: demographic characteristics, including intellectual ability assessed using the “Kyoto University NX-15” IQ test; head MRI data; and results of cognitive tasks and questionnaires^39^. From the 600 initial enrollees, 564 participated in the initial wave, and because of subsequent dropouts, 409 participants were able to provide complete responses to the scales used in this study. Of the 409 participants, nine participants who self-reported a history of treatment for neurological or psychiatric disorders were excluded in the pre-analysis phase. Thus, a total of 400 participants were analyzed in this study. Participants took part in the experiment according to the following schedule: demographic data collection and IQ test, May 17–July 22, 2012; MRI scanning, October 6, 2012–February 16, 2013; social cognitive function test, April 27–June 22, 2013; and evaluation of perceived child-rearing attitudes, September 2–October 26, 2013. This study was approved by the Ethics committees of Tamagawa University (TRE18-030) and Graduate School of Tokyo Medical and Dental University (M2020-079). The study protocols complied with the Declaration of Helsinki, and all participants provided informed consent.

### Evaluation of perceived child-rearing attitudes of parents

The nurturing environment was assessed using the Japanese version of the short form of Egna Minnen Beträffande Uppfostran (s-EMBU)^69,70^. The s-EMBU is a self-administered psychological questionnaire in which respondents reflect on how their parents nurtured them during their childhood. It comprises a 4-point Likert scale (1-Never, 2-Sometimes, 3-Often, 4-Always) with 23 items each for the father and mother; the items are subcategorized as rejection (seven items), emotional warmth (six items), overprotection (nine items), and unclassified (one item) items. The mean score for “rejection” was used as an indicator of abuse, since prior research showed that EMBU-rejection scores predict scores for physical abuse, physical neglect, and psychological neglect on the Childhood Trauma Questionnaire (CTQ). EMBU-emotional warmth is known to be inversely related to psychological neglect on the CTQ, but its coefficient in the regression analysis is smaller than that of rejection. Therefore, we focused our analysis on rejection^67,71^. Since the attachment formation in a child is considered to be mother-dominant up to adolescence^72^, we hypothesized that the effects of paternal and maternal rejection on a child's neurodevelopment might differ. We analyzed paternal, maternal, and parental rejection (their average), separately. The score ranges from 1 to 4. As the score is the average of the score of the seven items on the Likert scale, it is treated as a continuous variable^73^.

### Evaluation of social cognitive function

Social cognitive function was assessed using a video-based facial expression recognition task called the “Fake Smile Detection Task,” which is published on the webpage BBC-Spot the Fake Smile^74^ and has been used to examine social cognitive function in non-patient adults and children^75–77^. The task involved watching 20 different 4-s videos and determining whether the person’s smile in the video was “genuine” or “fake.” A genuine smile or the Duchenne smile has been defined as a smile that not only lifts the edges of the lips but also involves movement of the orbicularis oculus muscle and is known to be associated with enjoyment and other positive emotions^78^. On the other hand, a fake smile or a fabricated smile is defined as "a smile that is intentionally created to convince others that you are enjoying yourself when there is no such enjoyment existing" and in which the orbicularis oculi muscles are not active^79^. The videos changed from neutral to smiling expressions over 4 s, with 10 videos each having genuine and fake smiles. The task was accompanied by two preliminary questions about participants' views of life in general (optimism–pessimism) and their confidence in answering the task correctly, but these were not used in the analysis; only the number of correct answers in this task was analyzed.

### Head MRI scanning

MRI scans were performed using a 3-Tesla MRI scanner (Siemens Trio A Tim, Erlangen, Germany). High-resolution anatomical images were acquired using a T1-weighted 3D magnetization prepared rapid acquisition gradient echo sequence (repetition time = 2000 ms, echo time = 1.98 ms, field of view = 256 × 256 mm, number of slices = 192, voxel size = 1 × 1 × 1 mm). T1-weighted MR images were segmented using the FreeSurfer package (version 5.33.0 for Linux CentOS, http://surfer.nmr.mgh.harvard.edu/, USA) to determine the hippocampal and amygdala volumes and intracranial volume. We used the hippocampal volume:intracranial volume ratio (HpVR) and the amygdala volume:intracranial volume ratio (AmVR) to correct the intracranial volume.

### Statistical analysis

Descriptive analyses were performed to examine demographic and clinical variables. All variables were checked for normality of distribution using the Kolmogorov–Smirnov test (Lilliefors test). Correlations between the variables HpVR, AmVR, EMBU rejection score; number of correct responses to the Fake Smile Detection Task; and covariates of age, sex, and IQ were plotted into a scatterplot matrix. Spearman's rank correlations were then obtained for all variables. Differences by sex were tested with the Mann–Whitney U test. Subsequently, SEM analysis was used to determine whether hippocampal volume mediates the effect of maternal rejection on Fake Smile Detection Task score. With the significant effect of the interaction term between maternal rejection and age on hippocampal volume, we hypothesized that the main results would be affected by age. Therefore, we divided the participants into two groups using mean age and performed a post hoc SEM analysis. Since some variables were skewed, as shown in Table 1, we calculated the confidence intervals using the bootstrap method (10,000 iterations) in all analyses^80^. Statistical analyses were performed using IBM (NY, U.S.A.) Statistical Package for the Social Sciences version 27. The analyses using SEM were performed by IBM Amos version 27.0.0.

### Supplementary Information

Supplementary Tables.

## Data Availability

The datasets analyzed during the current study are not publicly available because they contain participants' confidential information, but are available from the corresponding author upon reasonable request.
