# Supplementary material for: Hippocampal volume mediates the relationship of parental rejection in childhood with social cognition in healthy adults
Source: Sci Rep. 2023 Nov 6;13:19167. doi: 10.1038/s41598-023-46512-2 (PMC10628272; doi:10.1038/s41598-023-46512-2)
Supplement: Supplementary file 1 — Supplementary Tables. [file 41598_2023_46512_MOESM1_ESM.docx]

**Supplementary data**

Supplementary Table S1. Summary of coefficients from SEM results with the bootstrapping procedure

|  | | |  |  |  |  | **Bootstrapped** |
| --- | --- | --- | --- | --- | --- | --- | --- |
| **Variables** | | | **B** | **SE** | **β** | **t** | **p** |
| HpVR | <--- | Sex | −.016 | .006 | −.133 | −2.851 | .004** |
| HpVR | <--- | Age | −.002 | .000 | −.263 | −5.573 | <.001** |
| HpVR | <--- | Maternal rejection | −.026 | .007 | −.188 | −3.978 | <.001** |
| HpVR | <--- | Maternal rejection*Age | .002 | .001 | .123 | 2.616 | .009** |
| AmVR | <--- | Sex | .007 | .003 | .114 | 2.405 | .020* |
| AmVR | <--- | Age | −.001 | .000 | −.277 | −5.791 | <.001** |
| AmVR | <--- | Maternal rejection | −.010 | .003 | −.142 | −2.962 | .002** |
| AmVR | <--- | Maternal rejection*Age | .000 | .000 | .027 | .573 | .538 |
| FSDT score | <--- | HpVR | 4.394 | 2.053 | .108 | 2.140 | .032* |
| FSDT score | <--- | Maternal rejection | −.524 | .281 | −.094 | −1.864 | .062 |
| FSDT score | <--- | Maternal rejection*Age | .009 | .025 | .018 | .352 | .711 |
|  |  |  |  |  |  |  | **Bootstrapped** |
| **Variables** | | | **r** |  |  |  | **p** |
| Age | <---> | Maternal rejection | −.122 |  |  |  | .016* |
| Age | <---> | Maternal rejection*Age | .060 |  |  |  | .228 |
| Maternal rejection | <---> | Maternal rejection*Age | −.086 |  |  |  | .086 |
| e2 | <---> | e3 | .550 |  |  |  | <.001** |

Variables Age, Maternal rejection, and Maternal rejection * Age are centered. e2 and e3 indicate errors. SEM, structural equation modeling; HpVR, hippocampal volume:intracranial volume ratio; AmVR, amygdala volume:intracranial volume ratio; FSDT, Fake Smile Detection Task; B, observed coefficients; β, standardized coefficients; r, correlation coefficients; **, p <.01; *, p <.05

Supplementary Table S2. Summary of coefficients from post hoc SEM results with the bootstrapping procedure

1. Younger group

|  | | |  |  |  |  | **Bootstrapped** |
| --- | --- | --- | --- | --- | --- | --- | --- |
| **Variables** | | | **B** | **SE** | **β** | **t** | **p** |
| HpVR | <--- | Sex | −.005 | .008 | −.040 | −.587 | .557 |
| HpVR | <--- | Maternal rejection | −.037 | .009 | −.289 | −4.236 | <.001** |
| AmVR | <--- | Sex | .013 | .004 | .207 | 2.978 | .003** |
| AmVR | <--- | Maternal rejection | −.007 | .005 | −.106 | −1.531 | .126 |
| FSDT score | <--- | HpVR | 8.540 | 3.119 | .198 | 2.738 | .006** |
| FSDT score | <--- | Maternal rejection | −.540 | .403 | −.097 | −1.339 | .181 |
|  |  |  |  |  |  |  | **Bootstrapped** |
| **Variables** | | | **r** |  |  |  | **p** |
| e2 | <---> | e3 | .542 |  |  |  | <.001** |

1. Older group

|  | | |  |  |  |  | **Bootstrapped** |
| --- | --- | --- | --- | --- | --- | --- | --- |
| **Variables** | | | **B** | **SE** | **β** | **t** | **p** |
| HpVR | <--- | Sex | −.029 | .008 | −.238 | −3.487 | <.001** |
| HpVR | <--- | Maternal rejection | −.011 | .010 | −.077 | −1.127 | .260 |
| AmVR | <--- | Sex | .001 | .004 | .014 | .207 | .836 |
| AmVR | <--- | Maternal rejection | −.011 | .005 | −.152 | −2.183 | .029* |
| FSDT score | <--- | HpVR | 1.029 | 2.786 | .026 | .369 | .712 |
| FSDT score | <--- | Maternal rejection | −.371 | .399 | −.066 | −.932 | .351 |
|  |  |  |  |  |  |  | **Bootstrapped** |
| **Variables** | | | **r** |  |  |  | **p** |
| e2 | <---> | e3 | .589 |  |  |  | <.001** |

e2 and e3 indicate errors. SEM, structural equation modeling; HpVR, hippocampal volume:intracranial volume ratio; AmVR, amygdala volume:intracranial volume ratio; FSDT, Fake Smile Detection Task; B, observed coefficients; β, standardized coefficients; r, correlation coefficients; **, p <.01; *, p <.05

Supplementary Table S3. List of papers published in this research project

| No. | Bibliographic information |
| --- | --- |
| 1. | Tanaka H, Shou Q, Kiyonari T, Matsuda T, Sakagami M & Takagishi H. Right dorsolateral prefrontal cortex regulates default prosociality preference. *Cereb Cortex*, bhac429. (2022). |
| 2. | Fermin ASR, Kiyonari T, Matsumoto Y. Takagishi H, Li Y, Kanai R, Sakagami M, Akaishi R, Ichikawa N, Takamura M & Yokoyama S. The neuroanatomy of social trust predicts depression vulnerability. *Sci Rep*, **12**, 16724. (2022). |
| 3. | Shou Q, Yamada J, Nishina K, Matsunaga M, Matsuda T & Takagishi H. Association between salivary oxytocin levels and the amygdala and hippocampal volumes. *Brain Struct Funct*, **227**, 2503-2511. (2022). |
| 4. | Shou Q, Yamada J, Nishina K, Matsunaga M, Kiyonari T & Takagishi H. Is oxytocin a trust hormone? Salivary oxytocin is associated with caution but not with general trust. *PLoS One*, **17**, e0267988. (2022). |
| 5. | Nishina K, Shou Q, Takahashi H, Sakagami M, Inoue-Murayama M & Takagishi H. Association between polymorphism (5-HTTLPR) of the serotonin transporter gene and behavioral response to unfair distribution. *Front Behav Neurosci*, **16**, 762092. (2022). |
| 6. | Yamada J, Nakawake Y, Shou Q, Nishina K, Matsunaga M & Takagishi H. Salivary oxytocin is negatively associated with religious faith in Japanese non-Abrahamic people. *Front Psychol*, **12**, 705781. (2021). |
| 7. | Ishihara T, Miyazaki A, Tanaka H, Fujii T, Takahashi M, Nishina K, Kanari K, Takagishi H & Matsuda T. Childhood exercise predicts response inhibition in later life via changes in brain connectivity and structure. *Neuroimage* **237**, 118196. (2021). |
| 8. | Nishina K, Takagishi H, Takahashi H, Sakagami M & Inoue-Murayama M. Association of polymorphism of arginine-vasopressin receptor 1A (AVPR1a) gene with trust and reciprocity. *Front Hum Neurosci*, **13**, 230. (2019). |
| 9. | Nishina K, Takagishi H, Fermin ASR, Inoue-Murayama M, Takahashi H, Sakagami M & Yamagishi T. Association of the oxytocin receptor gene with attitudinal trust: role of amygdala volume. *Soc Cogn Affec Neurosci*, **13**, 1091-1097. (2018). |
| 10. | Yamagishi T, Li Y, Fermin ASR, Kanai R, Takagishi H, Matsumoto Y, Kiyonari T & Sakagami M. Behavioural differences and neural substrates of altruistic and spiteful punishment. *Sci Rep*, **7**, 14654. (2017). |
| 11. | Yamagishi T, Matsumoto Y, Kiyonari T, Takagishi H, Li Y, Kanai R & Sakagami M. Response time in economic games reflects different types of decision conflict for prosocial and proself individuals. *Proc Natl Acad Sci U S A*, **114**, 6394-6399. (2017). |
| 12. | Yamagishi T, Takagishi H, Fermin ASR, Kanai R, Li Y & Matsumoto Y. Cortical thickness of the dorsolateral prefrontal cortex predicts strategic choices in economic games. *Proc Natl Acad Sci U S A*, **113**, 5582-5587. (2016). |
| 13. | Matsumoto Y, Yamagishi T, Li Y & Kiyonari T. Prosocial behavior increases with age across five economic games. *PLoS One*, **11**, e0158671. (2016). |
| 14. | Yamagishi T, Li Y, Matsumoto Y & Kiyonari T. Moral bargain hunters purchase moral righteousness when it is cheap: within-individual effect of stake size in economic games. *Sci Rep*, **6**, 27824. (2016). |
| 15. | Nishina K, Takagishi H, Inoue-Murayama M, Takahashi H & Yamagishi T. Polymorphism of the oxytocin receptor gene modulates behavioral and attitudinal trust among men but not women. *PLoS One*, **10**, e0137089. (2015). |
| 16. | Yamagishi T, Li Y, Takagishi H, Matsumoto Y & Kiyonari T. In search of homo economicus. *Psychol Sci*, **25**, 1699-1711. (2014). |
